# Identification of Sensory Processing and Integration Symptom Clusters: A Preliminary Study

**DOI:** 10.1155/2017/2876080

**Published:** 2017-11-16

**Authors:** Lucy Jane Miller, Sarah A. Schoen, Shelley Mulligan, Jillian Sullivan

**Affiliations:** ^1^STAR Institute, 5420 S. Quebec Street, Suite 105, Greenwood Village, CO 80111, USA; ^2^Department of Occupational Therapy, 4 Library Way, University of New Hampshire, Durham, NH 03824, USA; ^3^Northeastern University, 312E Robinson Hall, 360 Huntington Ave., Boston, MA 02115, USA

## Abstract

**Rationale:**

This study explored subtypes of sensory processing disorder (SPD) by examining the clinical presentations of cluster groups that emerged from scores of children with SPD on the Sensory Processing 3-Dimension (SP-3D) Inventory.

**Method:**

A nonexperimental design was used involving data extraction from the records of 252 children with SPD. Exploratory cluster analyses were conducted with scores from the SP-3D Inventory which measures sensory overresponsivity (SOR), sensory underresponsivity (SUR), sensory craving (SC), postural disorder, dyspraxia, and sensory discrimination. Scores related to adaptive behavior, social-emotional functioning, and attention among children with different sensory modulation patterns were then examined and compared.

**Results:**

Three distinct cluster groups emerged from the data: High SOR only, High SUR with SOR, and High SC with SOR. All groups showed low performance within multiple domains of adaptive behavior. Atypical behaviors associated with social-emotional functioning and attention varied among the groups.

**Implications:**

The SP-3D Inventory shows promise as a tool for assisting in identifying patterns of sensory dysfunction and for guiding intervention. Better characterization can guide intervention precision and facilitate homogenous samples for research.

## 1. Introduction

Current estimates indicate that 5% to 16.5% of the general population [1, 2] have symptoms associated with sensory processing challenges and these estimates are higher for clinical populations such as autism spectrum disorder (ASD) [3] and attention deficit hyperactivity disorder (ADHD) [4]. Sensory processing is part of normal development and reflects one's ability to interpret and respond to daily sensory experiences [5]. The integration of sensory information contributes to successful functioning in daily life reflected in our ability to self-regulate, interact socially [2], and develop adaptive behavioral skills/abilities [6]. Thus, sensory processing is only considered a problem when it interferes with functioning in daily life. In fact, the autism literature has been recently characterized as a form of atypical sensory processing that enhances daily life called enhanced perception linked to superior sensory acuity [7]. However, some subtypes of sensory processing have been associated with impairments in activities of daily living [6] and with other kinds of behavioral problems [8]. An increase in recognition of sensory processing challenges [7] supports the need to articulate patterns of dysfunction and associated functional deficits across clinical populations. Clinically meaningful descriptions will facilitate increased understanding of the clinical presentation, which can provide more specificity for guiding intervention and greater homogeneity of samples for research.

Interest in understanding and defining the patterns of sensory processing dysfunction has been prevalent since Ayres first developed the Southern California Sensory Integration Tests [9], followed by the Sensory Integration and Praxis Tests [SIPT] [10]. With the publication of the sensory profile [11], responsivity patterns characteristic of individuals with sensory modulation challenges were included. Davies and Tucker [12] summarized the evidence from 1986 to 2006, and they found that the literature was limited by the specific assessment tools used because no single study included a comprehensive assessment of sensory function and sensory-based motor performance. Thus, it is difficult to arrive at a consensus regarding factors or cluster groups across studies due to the varied constructs evaluated by different assessment tools, as well as due to heterogeneity and comorbidities in the samples studied [13].

Sensory processing dysfunction includes a heterogeneous set of symptoms that affects the manner in which individuals use sensory information for emotion regulation, motor performance, social interaction, and daily life functioning at home, at school, and in the community [14–16]. Some research to date has described the neurodevelopmental, behavioral, and functional characteristics and correlates of children who present with sensory processing challenges. For example, a systematic review by Koenig and Rudney [17] found that children with sensory overresponsive (SOR) patterns and sensory-related motor dysfunction have more difficulty with social participation and performance of activities of daily living than children with sensory under-responsivity (SUR). Reynolds and Lane [18] also found SOR to be associated with poor performance of activities of daily living. Elbasan et al. [19] associated disruption in performance in activities of daily living with visual and tactile discrimination problems, postural problems, and dyspraxia.

SOR in the tactile and auditory domains has been given the most attention in the literature. SOR has been associated with anxiety in children and adolescents previously classified as Asperger's syndrome (e.g., high functioning autism) [20] as well as with problems acquiring a number of adaptive behavior and functional skills [18, 21, 22]. SOR has also been associated with children who have attention deficit disorders [17, 22–24]. Although we are beginning to understand the clinical presentations of each sensory processing pattern in various clinical conditions, there is a need for further clarification of relations among patterns and differential association with occupational performance in children without comorbid diagnoses [12].

This study fills the gap in research elucidated by Davies and Tucker [25] by using a comprehensive assessment of sensory processing functions including sensory modulation, sensory discrimination, and sensory-based motor abilities, called the Sensory Processing 3-Dimension Inventory [26], previously called the Sensory Processing Scale [27]. We use a more homogeneous sample, since samples with different diagnoses and comorbidities may present differently, and our investigation of patterns includes constructs related to functioning in everyday life. Thus, the present study expands upon previous studies by examining characteristics within sensory modulation dysfunction related to adaptive behavior, social-emotional functioning, and inattention/overactivity; the study by Mailloux and colleagues [28] examined patterns of dyspraxia and sensory discrimination and the study by Mailloux and colleagues [28, 29] only examined sensory modulation. Knowledge of the patterns of dysfunction and associated functional impairments provides greater homogeneity of samples in research studies which allows for comparisons of results across studies [12]. Clinicians also need this information to increase treatment specificity and communication among parents, teachers, and other professionals.

Cluster analysis was used to determine whether a sample of children with known sensory processing challenges could be organized into clinically meaningful groups based on similarities and differences. Cluster analyses are exploratory, multivariate data reduction strategies that are commonly used to discover groupings of individuals across a variety of clinical conditions that tend to be heterogeneous in nature [30]. Cluster analysis facilitates the identification of patterns within groups and is useful in evaluating diagnostic taxonomies [31, 32]. Information regarding patterns of dysfunction is helpful so that interventions can be tailored to meet specific sensory processing and associated functional challenges of each group identified. Researchers can also use information from cluster analyses to form more homogeneous samples in treatment effectiveness studies and when investigating the underlying neurological mechanisms of a disorder. The following research questions were addressed: (a) what distinct and clinical meaningful cluster groups emerge based on scores from the SP-3D Inventory? and (b) what are the characteristics of children within each of the cluster groupings with respect to dimensions adaptive behavior, social-emotional functioning, and inattention/overactivity?

## 2. Method

### 2.1. Procedures and Participants

The study was conducted at Sensory Therapies And Research (STAR) Institute for Sensory Processing Disorder in Greenwood Village, CO, and involved retrospective data extraction from the charts of clients with SPD seen from 2007 to 2013. The data collected were from caregiver report measures used in routine clinical care. Chart review and data entry of deidentified information were completed by research assistants, supervised by a senior researcher following procedures approved by the Rocky Mountain University of Health Professions Institutional Review Board.

The sample consisted of data from 252 children ages 4 to 14 years (*M* = 6.9 years, SD = 2.05) who did not meet criteria for an autism spectrum disorder, a neurologic or orthopedic syndrome, or a mental health diagnosis based on parent report of medical history from a community pediatrician. Males made up 180 of the 252 participants (71.4%) and 80% of the sample were Caucasian. Socioeconomic status was based on parent education, with 61% of parents reporting that they had a high school education and 39% a college education or above. All participants completed a 2-hour, comprehensive performance-based occupational therapy evaluation including a standardized assessment of motor functioning, norm-referenced parent-rating scales of sensory, adaptive, and problem behaviors, and clinical observations of sensory processing, postural control, and motor performance. Only those children who had complete data on the Sensory Processing Three-Dimension Inventory were included. This sample was representative of a clinical sample of children with SPD referred to the STAR Institute for sensory and behavioral challenges that significantly interfered with daily functioning at home, at school, and/or in the community.

### 2.2. Instrumentation

*Sensory Processing Three-Dimension Inventory*. The Sensory Processing Three-Dimension (SP-3D) Inventory [33, 34] utilizes parent report to measure all subtypes of SPD based on the nosology [13], with six subscales: sensory overresponsivity (SOR), sensory underresponsivity (SUR), sensory craving (SC), postural disorder (PD), dyspraxia (DYS), and sensory discrimination disorder (SDD). For the three modulation subscales (SOR, SUR, and SC) and one sensory discrimination subscale, items address behaviors believed to be associated with the processing of the input from the visual, tactile, vestibular, proprioception, auditory, gustatory, and olfactory sensory domains. Scoring utilizes a binary system which requires the informant to indicate whether any of the behavioral descriptions/items apply to their child (applicable = 1; not applicable = 0). The SP-3D Inventory takes approximately 20 minutes to complete and consists of 182 items. Total scores reflect the sum of items endorsed by the parent on each subscale or SPD subtype, and higher scores suggest greater impairment. Scores for this study were transformed into percentages of total items endorsed to account for the variability in total number of items on each subscale, which were as follows: SOR-47 items; SUR-21 items, SC-28 items, SDD-26 items; posture disorder-23 items; and dyspraxia-37 items. Previous research demonstrates acceptable internal consistency reliability (*α* = .63–.75) and discriminative validity (Cohen's *d* = .56–1.53 [33, 35]. Principal axis factor analysis also confirmed the internal structure of the modulation subscales [33]. For this sample, internal consistency reliability for the subscales ranged from *α* = .80 to *α* = .92).

*Adaptive Behavior Assessment Scale-II.* The Adaptive Behavior Assessment Scale (ABAS-II) [36] is a norm-referenced, caregiver rating scale for individuals from birth to 21 years. The ABAS-II provides a comprehensive assessment of a child's adaptive behavior including functioning in ten related adaptive skill areas: communication, community use, functional academics, school/home living, health and safety, leisure, self-care, self-direction, social, and work. Three composite scores, conceptual, social, and practical, make up the General Adaptive Composite (GAC). Higher scores reflect* better* adaptive functioning. Internal consistency reliability for the GAC and all adaptive skill areas was found to be high for all age groups (*α* = .79–.99). Data in the test manual supports the tool's reliability and validity.

*Behavior Assessment System for Children-2.* The Behavior Assessment System for Children-2 (BASC-2) [26] is a norm-referenced measure of emotional and behavioral disorders for children 4 to 18 years. The parent-rating measure assesses a child's problem behaviors in the community and at home, including hyperactivity, aggression, conduct problems, anxiety, depression, somatization, attention problems, learning problems, atypicality, and withdrawal. The four composite scores are externalizing problems, internalizing problems, adaptive skills, and behavioral symptoms index. Higher scores indicate* greater* impairment. For this study, the adaptive subscale was not used because the content is similar to the ABAS-II, and the conduct disorder subscale score was not reported because the age range was limited to children 6 years and older. Reliability and validity data are reported to be strong including internal consistency reliability of the scales with Cronbach*α* values ranging from .88 to .94 [26].

*The Swanson, Nolan, and Pelham Parent-Rating Scale*. The Swanson, Nolan, and Pelham Parent-Rating Scale (SNAP-IV) [27] is an 18-item, parent report used to characterize ADHD features. It has two subscales characterizing each of the ADHD subtypes; 9 items represent the hyperactive/impulsive subtype, and 9 items represent the inattentive subtype. A 4-point Likert scale is used to rate behaviors from 0, not at all, to 3, very much. Internal consistency reliability for the parent report form is high*α* = .94 [30] with Cronbach *α* values for the inattentive and hyperactive/impulsive subscales .90 and .79, respectively [30]. Studies of predictive validity, sensitivity, and specificity support the tool as an acceptable measure of behaviors associated with ADHD [30].

### 2.3. Data Analysis

A Ward's agglomerative hierarchical cluster analysis was first conducted using the items from all subscales of the SP-3D Inventory to identify potential cluster solutions based on squared Euclidean values in a distance matrix [37]. Next, a* K*-means clustering algorithm based on the results from the hierarchical analysis (as described by [38]) was conducted to derive the final clusters. No meaningful cluster groupings emerged using all six of the SP3D Inventory subscales. Cluster analysis is considered an exploratory and somewhat subjective technique which evaluates children's scores on all variables by how close or distant they are from other children [39]. A lack of meaningful cluster groups may have occurred because of the inherent heterogeneity of children with SPD, that is, children having many different combinations of the various subtypes. To explore the data further with less variability, cluster analysis was repeated using only the scales representing sensory modulation behaviors. When there appears to be no interpretable solution, the literature suggests carefully considering the variables that are included in the analysis [39, 40]. Since sensory modulation has a strong theoretical foundation and is a common sensory processing symptomatology, cluster analysis was rerun using the three modulation subscales. A 3-cluster solution which grouped children based on the scoring of items on the SOR, SUR, and SC subscales best fit the data (based on effect size attributed to each cluster variable). Subsequent analyses then examined the specific characteristics of each cluster group, and differences in their scores from the ABAS-II, BASC-2, and SNAP-IV as well as from the other three SP3D Inventory subscales (e.g., posture, dyspraxia, and sensory discrimination).

One-way ANOVAs were performed to assess cluster group differences. Games-Howell post hoc tests were used when the Brown-Forsythe test for robustness of the median was significant. Due to the exploratory nature of this investigation, and to minimize the potential for Type II errors, an alpha level of *p* < 0.05 was used to determine statistical differences despite multiple comparisons, as suggested by [41].

## 3. Results

**Figure 1:**
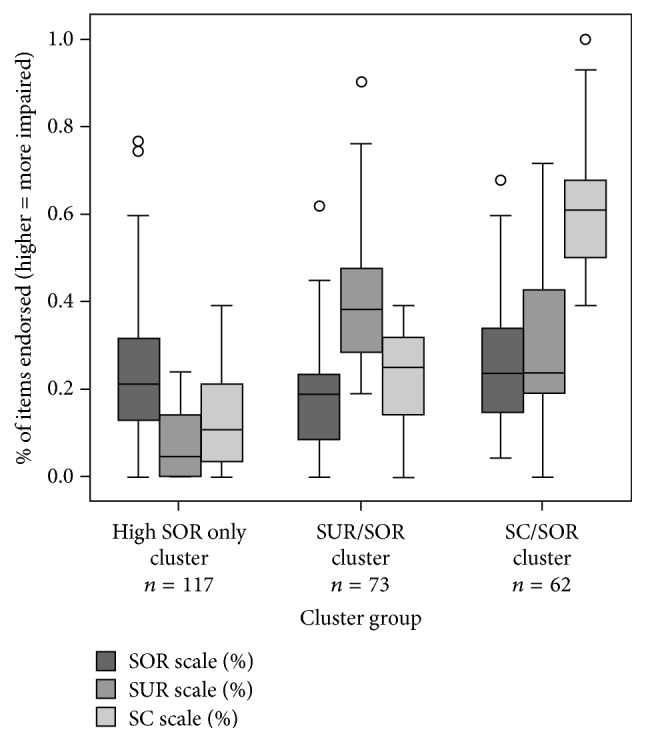
Cluster groupings.

The distance matrix from the hierarchical cluster analysis provided support for a three-cluster solution: (a) High SOR only cluster (*n* = 117); (b) High SUR plus SOR cluster (*n* = 73); and (c) High SC plus SOR cluster (*n* = 62) (see Figure 1).

In terms of demographics, the High SOR only group was 70.9% male with a mean age of 7.28 (SD = 2.23). The High SUR plus SOR (SUR/SOR) cluster group was 64.4% male with a mean age of 6.78 (SD = 1.92), and the High SC plus SOR (SC/SOR) cluster group was 80.6% male with a mean age of 6.37 (SD = 1.67). A one-way ANOVA confirmed cluster group differences for age,* F *(2, 249) = 4.37, *p* = 0.014, with the High SOR only cluster group being significantly older than the SC cluster group (*p* = 0.004) but not significantly different from the SUR cluster group (*p* = 0.099). Chi-square analysis showed no significant differences in gender distribution among the groups, *χ*^2^ (2) = 4.37, *p* = 0.11.

The three-cluster solution in the K-means analysis produced groups that significantly differed in their SP-3D Inventory SOR subscale score, *F* (2, 249) = 3.45, *p* = 0.033; *η*^2^ = .011, SUR subscale score, *F* (2, 249) = 142.89, *p* < 0.001, *η*^2^ = .376, and SC subscale score *F* (2, 249) = 326.93, *p* < 0.001, *η*^2^ = .724. Games-Howell post hoc tests showed that the High SC/SOR cluster group had more SOR behaviors than was found in the High SUR/SOR cluster group (*p* = 0.028) although the High SOR only cluster group did not significantly differ from the other cluster groups on their SOR subscale scores (*p* > 0.05). Thus, all cluster groups had some behaviors related to overresponsivity. Sensory craving behaviors were much more prevalent in the High SC/SOR cluster group and sensory underresponsivity subscale behaviors were more prevalent in the High SUR/SOR cluster group (*p* < 0.01).  Table 1 summarizes the findings for the SP-3D Inventory by cluster group.

Using one-way ANOVAs, significant group differences among the three-cluster groups were found on the SP-3D Inventory postural subscale,* F* (2, 249) = 7.33, *p* < 0.01; praxis subscale,* F *(2, 249) = 23.96, *p* < 0.001; and sensory discrimination subscale,* F* (2, 249) = 28.21, *p* < 0.001. Post hoc tests revealed that, in comparison with the High SOR only cluster group, the High SC/SOR and High SUR/SC groups showed significantly higher (more impairment) scores on the postural, praxis, and sensory discrimination disorder subscales (*p* < 0.01 for all). The High SUR/SOR and High SC/SOR cluster groups did not significantly differ from each other on the total scores for the postural, praxis, or discrimination subscales.

Data from the SP3D Inventory was available from a previous study [26] for a nonrepresentative typically developing sample of children (*n* = 140) for comparison to the clinical sample. All subscale scores for the High SUR/SOR and High SC/SOR cluster groups are more than two standard deviations above the typical mean. Subscale scores for the High SOR only group were most elevated for sensory overresponsivity. See Table 1.

An exploratory analysis was conducted of frequency of item endorsement across the three-cluster groups to discover trends within the data. Significant or near significant differences were identified for several items. The High SUR/SOR cluster group had more problems with dressing and undressing (*p* = 0.019) such as placing arm or leg correctly in clothing (*p* = 0.044), completing fasteners (*p* = 0.066), and tending to look disheveled (*p* = 0.012). They also tended to prefer sedentary activities (*p* < 0.001) and had difficulty climbing on or over objects (*p* = 0.010) and licking an ice cream cone (*p* = 0.067). The High SC/SOR cluster group tended to crave tactile stimuli (examining toys by touching and feeling; *p* = 0.082) and had more problems grading force needed for a task (*p* = 0.079), maintaining or copying rhythmical movements (*p* = 0.149), and differentiating printed figures that appear similar (*p* = 0.011).

The Adaptive Behavior Assessment System-II (ABAS-II) scores were available for 170 (67% of the sample) children, and they are summarized in Table 2. A one-way ANOVA showed no group differences on the General Adaptive Composite (GAC) nor any of the other composite scores (*p* > 0.1 for all). However, the mean GAC score was more than one standard deviation below the mean suggesting below-average functioning in adaptive behavior for all cluster groups. Scores on the conceptual composite (i.e., self-direction, functional academics, and communication) and the social composite (i.e., social skills and leisure activities) were at or below one standard deviation for the High SUR/SOR and High SC/SOR cluster groups and within normal limits for the High SOR only cluster group. The communication subscale of the ABAS-II showed significant differences* F *(2, 167) = 3.41, *p* = 0.035; among the groups with the High SUR/SOR cluster group scoring lower than the High SOR only group (*p* = 0.05). On the health and safety subscale, the High SUR/SOR group (*p* = 0.034) and High SC/SOR group (*p* = 0.06) had lower scores than the High SOR only group. Subscale mean scores for each of the three-cluster groups on self-direction, home living, and self-care were more than one standard deviation below the mean, while functional academics, communication, and leisure mean scores fell within the typical range for all three-cluster groups.

The Behavior Assessment System for Children-2 (BASC-2) scores were available for 188 of the subjects (74.6% of the total sample) and are presented in Table 3. For composite scores, one-way ANOVAs confirmed cluster group differences for externalizing behavior:* F *(2, 185) = 19.74, *p* < 0.001; internalizing behavior:* F *(2, 185) = 5.22, *p* = 0.006; and for the behavioral symptoms index:* F *(2, 185) = 15.63, *p* < 0.001. For externalizing, post hoc tests revealed that the High SC/SOR cluster group had more symptoms than the High SOR only and High SUR/SOR groups (*p* < 0.001; <0.002, resp.). For internalizing and externalizing composites, High SC/SOR cluster was the only cluster whose mean score fell in the clinically significant range, and this group had greater symptoms than both the High SUR/SOR group (*p* = 0.008) and the High SOR only group (*p* = 0.041). For the behavioral index composite, the High SUR/SOR and High SC/SOR cluster group's means fell in the clinically significant range. Both the High SUR/SOR cluster (*p* = 0.003) and the High SC/SOR cluster (*p* < 0.001) groups had greater symptoms than the High SOR only cluster group.

At the subscale level, Games-Howell post hoc tests revealed that hyperactivity showed the biggest difference between groups, with the High SC/SOR cluster showing the most symptoms compared to the High SUR/SOR cluster (*p* < 0.001) and the High SOR only group (*p* < 0.001). The High SOR only and High SUR/SOR groups differed but only marginally (*p* = 0.06). On the aggression subscale, only the High SC/SOR cluster group was in the clinically significantly range, while both the High SUR/SOR and High SC/SOR cluster groups had hyperactivity scores falling in the clinically significant range. The BASC-2 anxiety subscale showed that the High SC/SOR cluster group had the most symptoms, reaching statistical significance when compared to the High SUR cluster group (*p* = 0.006). The High SUR/SOR and High SOR only cluster groups did not differ on this subscale (*p* = 0.32). On the depression subscale, the High SC/SOR group again showed the most symptoms, with significant differences between the High SC/SOR group and the SUR (*p* = 0.018) and SOR (*p* = 0.004) groups. On the behavior atypicality and attention subscales, the High SOR only group had scores falling in the typical range, which was significantly different than the High SUR/SOR and High SC/SOR groups (*p* < 0.001 for both). The High SC/SOR and High SUR/SOR cluster groups did not significantly differ from each other on these scales. Withdrawal was in the clinically significant range for the High SUR/SOR cluster group only, and none of the groups had atypical conduct problems or somatization symptoms.

The SNAP-IV scores were available for 78 (31%) children in the sample with the results showing cluster group differences on the SNAP-IV total score,* F *(2, 76) = 24.49, *p* < 0.001. The High SC/SOR group had more symptoms on the SNAP-IV than the High SOR only (*p* < 0.001) and the High SUR/SOR (*p* = 0.009) groups, and the High SUR/SOR cluster had more symptoms than the High SOR only group (*p* = 0.002). In examining specific ADHD behaviors, post hoc tests indicated that the High SC/SOR cluster group had more hyperactivity symptoms than either the High SUR/SOR cluster group (*p* < 0.001) or the High SOR only cluster group (*p* < 0.001), but the High SC/SOR cluster group was not significantly different from the High SUR/SOR group on inattention (*p* = 0.942). The High SOR only group had fewer hyperactivity and inattention symptoms than the High SUR/SOR group (*p* = 0.044; *p* = 0.008, resp.) and fewer than the High SC/SOR group (*p* < 0.001; *p* = 0.003, resp.). Only fifty-five percent of this sample qualified for an ADHD diagnosis.

## 4. Discussion

Clusters that emerged from the analysis partially support the sensory modulation patterns, described by Miller et al. [13]. Although all three groups had symptoms of sensory overresponsivity, SUR and SC seemed to suggest distinct groups with the third group differentiated by elevated SOR symptoms only and fewer other sensory and motor symptoms, while distinct groups representing postural disorder, dyspraxia, and sensory discrimination disorder did not emerge from the analyses. Other methodology that has been used to examine sensory processing constructs was conducted by Su and Parham [42] using the Sensory Processing Measure [43], a similar measure of sensory processing challenges. In this study, Su and Parham used confirmatory factor analysis to test whether sensory questionnaire items represent distinct sensory processing constructs using a sample of 454 children from 2 to 10 years of age. They found that items associated with individual sensory systems, tactile, vestibular-proprioceptive, visual, and auditory systems, formed distinct factors rather than patterns of sensory processing such as sensory over- or underresponsivity and sensory discrimination.

Previous research has suggested that postural disorder and dyspraxia may be a single construct [44] and perhaps there is too much overlap in the SP-3D Inventory items from these two subscales to form distinct cluster groupings. Moreover, underlying sensory discrimination problems such as tactile discrimination has commonly been associated with motor planning deficits (called somatopraxis; see [28, 45]) which may be an explanation for the lack of sensory-based motor clusters emerging from this SP-3D Inventory data.

Upon examination of the characteristics of each of the sensory modulation cluster groups that did emerge, some differences were found related to postural, praxis, and sensory discrimination functions, adaptive behavior, and social-emotional and attention/hyperactivity behaviors. The High SOR only group had elevated overresponsivity scores but fewer symptoms related to postural disorder, dyspraxia, and sensory discrimination and all scores falling within normal limits on the BASC-2. The High SOR only group did have atypical scores on scales measuring performance in activities of daily living and self-direction. The High SUR/SOR group had many atypical adaptive behavior scores, with the lowest social composite score, similar delays in daily living skills and self-direction, and additional delays in health and safety and community use. Dyspraxia and postural symptoms were common in this group especially with a preference for sedentary activities and poor participation in dressing, undressing, and climbing. Inattention and withdrawal also scored high in this group. The High SC/SOR group had the greatest number of adaptive behaviors and behavioral symptoms and was differentiated from the other groups by clinically significant externalizing and internalizing behaviors, specifically hyperactivity, aggression, depression, and anxiety. The High SC/SOR cluster showed problems related to grading force, performing rhythmical movements, and visual discrimination. In adaptive behavior, the High SC/SOR group showed symptoms within all three composite scores measuring social, conceptual, and practical daily living skill domains.

A previous study of sensory modulation dysfunction phenotypes by James et al. [29] supported two of the cluster groups, SC and SUR, that were found in our study. Similarly, defining characteristics of the SC cluster included aggression, inattention, and many externalizing behaviors. Additionally, SOR was present in both clusters identified by James et al. [29] as it was in this study. Our results also support the coexistence of sensory modulation symptoms with motor problems, building on findings in previous studies [28, 29]. Clinically, it is not surprising that the SUR and SC cluster groups had associated motor difficulties as children who crave sensory input tend to engage in a great deal of nonproductive, nonpurposeful movement.

The finding that children fitting the High SC/SOR cluster also often have praxis problems may dispel the notion that sensory craving is just a compensatory behavioral strategy that is employed to counteract sensory over- or underresponsivity. Other evidence supporting our results [18, 46] suggests that SC comprises its own symptom cluster which can be associated with sensory-based motor challenges and externalizing behaviors. For the High SUR/SOR cluster, we suggest that the lack of awareness to body sensations coupled with a reduced or slowed reactivity to external environmental sensory demands affects their motor skill acquisition and motor planning.

The High SUR/SOR and High SC/SOR cluster groups had more symptoms in social-emotional and fewer adaptive behavior abilities than the High SOR only cluster group, although all three-cluster groups had challenges in adaptive behavior. Individuals in the High SC/SOR cluster group tended to be more aggressive, hyperactive, anxious, and depressed than individuals in the other two cluster groups. We found more attention problems in the High SUR/SOR and High SC/SOR cluster groups unlike a previous study that found an association between inattention and only sensory overresponsivity [28, 29].

Of interest is that children in the High SC/SOR cluster were slightly younger than the other two cluster groups. One possibility is that individuals with SC tend to be more disruptive and thus may be identified at an earlier age. Children with SUR are usually quiet and passive and may not be identified until daily life requirements (functions) at school or at home become intolerable. Another possibility is that sensory craving or externalizing behavior tends to decline as children with age, similar to the decline in hyperactivity that tends to occur in children with ADHD as they get older [47].

Sensory processing challenges are increasingly recognized as a component of other disorders such as ADHD [24, 48] and ASD [49, 50]. Studies aiming to describe phenotypic characteristics may assist in differentiating clinical conditions. Research describing sensory patterns in ASD suggest cluster groups based on sensory domain involvement such as tactile, auditory, and movement sensitivity [32]. Other studies suggest patterns based on severity of symptoms as well as reflecting the cooccurrence of hyperresponsiveness (e.g., SOR) and hyporesponsiveness (e.g., SUR) responsivity possibly similar to the SUR/SOR cluster group identified in this study [51, 52]. Furthermore, behavioral manifestations of sensory symptoms in ASD have been shown to be more severe than those in individuals without a comorbid diagnosis [53].

This study suggests that ADHD and children with sensory processing challenges have some characteristics in common but also may differ. Research examining ADHD symptoms within individuals with sensory processing challenges shows an overlap with behaviors such as hyperactivity, inattention, and motor incoordination cooccurring with sensory issues [54]. In this study, many children who did not meet criteria for an ADHD diagnosis fell within each of the clusters. Similarly, Ben-Sasson et al. [55] identified a group of children with elevated ADHD symptoms* only* as well as a group with elevated SOR symptoms* only,* thus supporting the independence and uniqueness of each disorder.

Delineation of patterns of sensory processing dysfunction will help to facilitate outcomes research because in order to compare the effectiveness of intervention approaches, homogenous samples are necessary [12]. The clusters identified in this study represent different symptom presentations which likely require differing intervention strategies, so a greater understanding of the distinguishing characteristics of children within each pattern will help guide intervention. Finally, clarifying various patterns of dysfunction sets the stage for the study of the neuroanatomical and neurophysiological underpinnings of these cluster groups [56].

Limitations within the study design must be considered in the interpretation of findings. First, data were collected retrospectively from just one private clinic in Colorado and therefore may not be representative of all clinical samples of children with sensory processing challenges. The BASC-2, ABAS-II, and SNAP-IV were available only for a subset of the participants resulting in smaller samples of children with data from these questionnaires. These findings should be replicated in other clinic settings and with different samples. The age range in this study was broad, so that if developmental changes exist within sensory symptom presentation, an examination of scores by age may yield different outcomes. Finally, the SP-3D Inventory is a new tool, and extensive study of its reliability and validity is needed to support its use as a comprehensive parent report measure of sensory processing. Future studies should focus on delineation of sensory domain characteristics of overresponsivity within the cluster groups to determine if this helps to further define the cluster groupings. Additionally, correlational studies are suggested to help determine if having both SUR and SOR or both SC and SOR put children at greater risk for behavioral and adaptive behavior problems compared to SUR or SC alone.

## 5. Conclusions and Implications for Occupational Therapy Practice

This study provides information to the categorization of patterns of children with sensory processing challenges who do not meet criteria for other clinical diagnoses. This work assists in enhancing the clarity of communication used to describe children with sensory processing challenges both within and outside the occupational therapy profession. The results supported three distinct groups all with symptoms of sensory overresponsivity (e.g., High SOR only, High SUR/SOR cluster, and High SC/SOR cluster), and within these patterns, differing symptoms related to motor challenges and sensory discrimination challenges were found. All three-cluster groups had some challenges in occupational performance with individuals in the High SUR/SOR and High SC/SOR clusters displaying more challenges than children in the High SOR only group. This study builds on previous work by further elucidating patterns of sensory processing dysfunction and associated functional and behavioral symptoms that characterize each group. A greater understanding of the strengths and challenges inherent within each pattern is useful for guiding the delivery of appropriate occupational therapy interventions and for selecting homogenous participants for research.

## Figures and Tables

**Table 1:** Mean scores from SP3D Inventory group.

Variable	SOR only group	SUR/SOR group	SC/SOR group	Typical group
	Mean (SD)	Mean (SD)	Mean (SD)	Mean (SD)
*SP3D Inventory*	*n* = 117	*n* = 73	*n* = 62	*n* = 140
SOR scale	10.6 (6.9)	8.89 (6.48)	11.95 (7.07)	2.25 (2.43)
SUR scale	1.73 (1.61)	8.42 (3.08)	6.35 (3.94)	0.96 (2.13)
SC scale	3.53 (3.18)	6.33 (3.05)	17.03 (4.13)	0.58 (1.14)
Posture scale	4.09 (4.29)	6.22 (5.05)	6.6 (5.35)	0.64 (1.91)
Praxis scale	7.49 (6.26)	14.23 (8)	12.15 (6.41)	1.57 (3.36)
Discrimination scale	1.87 (2.16)	4.1 (3.25)	4.74 (2.91)	0.48 (1.65)

**Table 2:** Mean scores from the ABAS II by cluster group.

Variable	SOR only group	SUR/SOR group	SC/SOR group
	Mean (SD)	Mean (SD)	Mean (SD)
*ABAS II*	*n* = 72	*n* = 49	*n* = 49
General Adaptive Composite	82.1 (16.2)	79.0 (17.1)	77.8 (12.6)
Conceptual composite	87.2 (15.4)	83.7 (15.9)	83.1 (12.7)
Communication	8.8 (3.2)	7.4 (3.3)	7.6 (3.3)
Functional academic	8.5 (3.3)	8.3 (3.5)	8.3 (2.6)
Self-direction	6.3 (3.6)	6.3 (3.1)	5.7 (2.5)
Social composite	88.3 (16.6)	82.9 (18.1)	84.7 (14.1)
Leisure	8.6 (3.0)	7.6 (3.3)	7.9 (2.8)
Social	7.1 (3.3)	6.6 (3.5)	6.9 (3.0)
Practical composite	79.6 (16.9)	75.9 (17.5)	75.9 (12.1)
Community use	7.6 (3.5)	7.1 (3.3)	6.9 (3.1)
Home living	6.4 (3.5)	5.9 (3.2)	5.8 (2.5)
Health and safety	8.3 (3.5)	6.6 (3.6)	6.9 (2.9)
Self-care	5.1 (2.7)	5.1 (2.9)	4.8 (2.0)

*Note*. Atypical on ABAS-II composite scores is ≤85. Atypical on ABAS-II subscales is ≤7.

**Table 3:** Mean scores from BASC-2 by cluster group.

Variable	SOR only group	SUR/SOR group	SC/SOR group
	Mean (SD)	Mean (SD)	Mean (SD)
*BASC-2*	*n* = 78	*n* = 57	*n* = 53
Externalizing composite	54.1 (10.5)	58.9 (11.4)	66.4 (11.5)
Hyperactivity	56.9 (11.3)	61.4 (11.2)	72.7 (12.0)
Aggression	52.6 (10.1)	56.7 (12.4)	61.2 (21.1)
Conduct problems	49.3 (13.1)	55.0 (11.9)	53.0 (9.3)
Internalizing composite	57.0 (11.9)	55.3 (11.6)	62.4 (12.8)
Anxiety	56.8 (13.0)	52.6 (11.5)	60.4 (14.2)
Depression	57.4 (11.9)	58.2 (11.4)	64.8 (13.4)
Somatization	51.9 (13.0)	52.7 (12.2)	53.8 (12.2)
Behavioral index composite	58.1 (10.2)	64.2 (10.4)	68.7 (12.2)
Atypicality	55.5 (11.1)	65.7 (14.3)	65.4 (16.0)
Withdrawal	58.1 (13.9)	60.2 (15.3)	56.5 (15.1)
Attention	56.0 (10.9)	62.4 (7.5)	64.6 (8.6)

*Note*. Atypical on BASC-2 is ≥60.
